# Shear Energy Evolution and Fracture Behavior of Rock–Concrete Interfaces Under Different Stress-Level Conditions

**DOI:** 10.3390/ma18040795

**Published:** 2025-02-11

**Authors:** Taoying Liu, Min Tang, Ping Cao, Mengyuan Cui, Longjun Dong

**Affiliations:** School of Resources & Safety Engineering, Central South University, Changsha 410083, China

**Keywords:** rock–concrete interface, direct shear test, stress level, energy evolution, fracture behavior

## Abstract

Indoor direct shear tests under different stress levels were conducted on sandstone–concrete samples to investigate the rock–concrete interfaces’ shear energy evolution features and fracture behaviors under different normal stresses, combined with acoustic emission (AE) and digital image correlation (DIC) techniques. The research results show that the growth of normal stress restricts the coalescence and failure of micro-cracks inside the sample and improves the bearing capacity. The shear strength of the sandstone–concrete cemented interface increases by 12.3–34.34% with increasing normal stress. The evolution behaviors of the total input energy, elastic strain energy and dissipated energy density are similar under different normal stress conditions, and the increase in normal stress raises the energy storage capacity of the sample, as well as the input external energy required for a sample’s failure, thereby enhancing the bearing capability of the sample. In addition, the AE count and *b* value characteristics indicate that crack propagation shows a three-stage variation trend. It can be seen from the RA (rise time/amplitude)-AF (AE count/duration time) curves that as the normal stress increases, the proportion of shear cracks in the sample progressively increases. When the final overall failure of the sample is imminent, the high-energy level fracture type changes from tensile fracture to shear fracture with increased normal stress, leading to an increasing percentage of shear fracture. Finally, the speckle results indicate that the nucleation and coalescence of tensile wing-shaped cracks are the main causes of sample failure. Under relatively high normal stress conditions, the damage degree of the serrated interface increases and the crack morphology becomes more intricate.

## 1. Introduction

Rock–concrete interfaces are relatively weak surface layers that broadly exist across diverse instances of geotechnical engineering, such as rock tunnels, concrete dams, arch bridge foundations, retaining walls, nuclear waste repositories, and rock-socketed piles [1,2,3,4,5,6]. In these cases, concrete is often used as a supporting material for rock engineering protection. When concrete is sprayed or poured on a rock mass surface, a cohesive interface is formed between the concrete and the rock foundation through cementitious bonding. The bonding surface represents the interface, or a discontinuity between two materials with distinct mechanical properties, which frequently becomes a potentially weak plane prone to instability and fracture, thereby affecting the safety and structural integrity of the overall engineering system [7]. In practical engineering structures, rock and concrete are often subjected to various complex external load disturbances, mainly in the form of shear loads. When shear loads act on the rock–concrete interface, they may precipitate interface damage and failure, resulting in slip instability and other forms of shear-induced failures. Ultimately, the safety and stability of the entire engineering structure are undermined. Therefore, investigations into the shear characteristics and failure properties of rock–concrete interfaces are considered to be of great practical significance.

In recent years, many scholars have conducted a series of shear tests on rock–concrete interfaces and obtained fruitful results, including the interfacial shear mechanisms [8,9,10,11,12], damage characteristics [13,14], and fracture mechanical properties [15,16,17]. For example, Hou et al. [8] conducted direct shear tests to investigate the influence of the initial normal stress and normal stiffness on the interface shear mechanism and strength under the condition of constant normal stiffness (CNS). Combining the Patton shear model with the upper-bound and lower-bound theorem of limit analysis, they proposed a regular triangular interface shear model under CNS conditions. And they found that roughness and material properties progressively impacted the shear behavior of the interface. Liu et al. [9] carried out a series of experimental studies on the shear characteristics of regular mudstone–concrete interfaces under different normal stiffnesses and different initial normal stresses. The results showed that there was an obvious compression phenomenon of the rough bodies in the mudstone during the shear process, and that the compression of the rough bodies would, to a certain extent, reduce the shear strength of the interface. Moreover, they also established a shear model suitable for the strength analysis of mudstone–concrete interfaces based on the Patton shear model, and verified its feasibility. In addition to analyzing via experiments and theories, some scholars have also utilized numerical methods to study the shear properties of rock–concrete interfaces [10,11,12]. To investigate the influence of bonds and the roughness of rock–concrete interfaces on the interface strength under low normal loading, Badika et al. [10] conducted a comparative study on the shear behaviors of smooth interfaces, rough artificial granite interfaces, and natural granite interfaces under low normal loading by combining the FEM software ABAQUS and direct shear tests. They observed that the cohesion formed on the smooth interfaces was negligible. The bonding strength of rough artificial granite interfaces was higher than that of natural interfaces, and the shear strength of the interface increased as the roughness increased. Saadat et al. [12] established a shear model for the interface with regular triangular rough bodies through PFC2D software. The simulation results showed that both the peak and residual strengths of the interface increased with an increase in the inclination angle of the rough bodies. Qiu et al. [16] studied the fracture characteristics of rock–concrete interfaces under impact loading and found that the crack propagation behavior of the interface was significantly affected by the loading rate. Yuan et al. [17] combined experiments with numerical simulation to study the fracture characteristics of rock–concrete interfaces. The results showed that the fracture energy, critical crack length, and double-K fracture parameter exhibited an apparent increasing trend with increasing roughness.

The preceding studies have significantly advanced the development of interfacial shear failure problems. However, according to the laws of thermodynamics, the deformation and destabilization of rocks under load are inevitably accompanied by energy conversion, and this evolution process can reflect the rocks’ failure more accurately [18,19]. The energy evolution of the interface is not only related to structural stability but also has a direct bearing on the long-term safety of engineering projects. Nevertheless, most of the current studies on the energy evolution of rock materials during the deformation and failure process mainly address compressive properties, including uniaxial and triaxial compression tests [20,21] and conventional and complex stress paths [22,23], as well as cyclic and fatigue loading conditions [24,25]. By contrast, the investigations into shear characteristics are relatively rare and mainly focus on pure rock structural surfaces or joints [26,27], and studies on energy evolution during the shear failure process of rock–concrete interfaces are even less numerous. Given this, we think it is necessary to carry out studies on the shear energy evolution of rock–concrete interfaces and explore the mechanism of interface energy accumulation, release, and transformation during the shear process, which plays a vital role in preventing potential engineering disasters.

As widely used non-destructive detection methods, acoustic emission (AE) and digital image correlation (DIC) techniques are frequently applied when conducting in-depth research on the mechanical performance and fracture characteristics of rocks [28,29]. Dong et al. [30] carried out expansion rupturing tests and elastic wave attenuation experiments and classified rock micro-fracture types by the RA and AF values of acoustic emission parameters. Based on Brazilian splitting tests of granite, Gan et al. [31] investigated the variation laws of the distribution of RA and AF values over time and explored the criteria for distinguishing between shear cracks and tensile cracks. Chmel et al. [32,33] compared the differences in AE characteristics and b values of granite under compression and impact failure to study the influence of the loading rate on rock damage. Guo et al. [34] conducted Brazilian splitting tests on rock–concrete cracked straight-through Brazilian disk (CSTBD) samples after thermal treatment, and utilized DIC techniques to explore the crack propagation process and fracture behavior. Guo et al. [35] performed four-point shear (FPS) tests for rock–concrete samples and monitored their fracture process by AE and DIC techniques. And, based on a machine learning algorithm, they processed the AE signals and quantitatively identified the damage types at the interfaces. In light of the existing studies, we employ DIC and AE techniques to comprehensively analyze the shear behaviors of the interfaces in shear tests conducted on rock–concrete interfaces, aiming to reveal the failure mechanism of the interfaces more accurately and assess their stability and safety.

To sum up, a typical sandstone was selected for preparing the sandstone–concrete specimens in this paper, and a series of direct shear tests were performed under different normal stress constraints, mainly aiming to describe and reveal the energy transformation and dissipation mechanism of interfaces in the shear process. At the same time, through the use of AE and DIC techniques, this paper comprehensively and deeply studies the trends of shear mechanical properties, instability characteristics, and fracture behaviors of the sandstone–concrete interface with stress levels. The test results provide a valuable theoretical reference for exploring the shear behaviors of rock–concrete interfaces, and are of guiding significance for the design and maintenance of engineering structures.

The subsequent sections of the paper are structured as follows: Section 2 presents the energy principles and the test procedure in this study, including the energy calculation methods and the experimental configuration. Section 3 analyzes the results of direct shear tests on the sandstone–concrete samples. It presents a clarification of the shear mechanical properties and energy evolution mechanism, as well as the brittleness evaluation of the sandstone–concrete samples based on energy evolution analysis. Section 4.1 and Section 4.2 present an investigation of AE characteristic parameters, including *b* values, AF, and RA. In addition, the temporal distribution characteristics of micro-fracture types in samples under different normal stress conditions are discussed in Section 4.3, followed by an analysis of the fracture behaviors of the interfaces in Section 4.4. The conclusions of this paper are presented in Section 5, as well as the limitations of this study and the required future research.

## 2. Energy Methodology and Test Program

### 2.1. Energy Calculation Methods

Based on the energy mechanism proposed by Xie et al. [18,19], the deformation and failure process of rocks under load is accompanied by energy input, accumulation, dissipation, and release. Assuming that during the deformation process of the rock mass element under external load, there is no heat exchange with the external environment, the total energy U generated by the work done by the external force is the actual energy absorbed by the rock sample. According to the first law of thermodynamics, we can obtain the equation

(1) $$U=U_{e}+U_{d}$$

where $U_{e}\;$ represents the unit releasable elastic strain energy and $U_{d}\;$ represents the unit dissipation energy.

**Figure 1:**
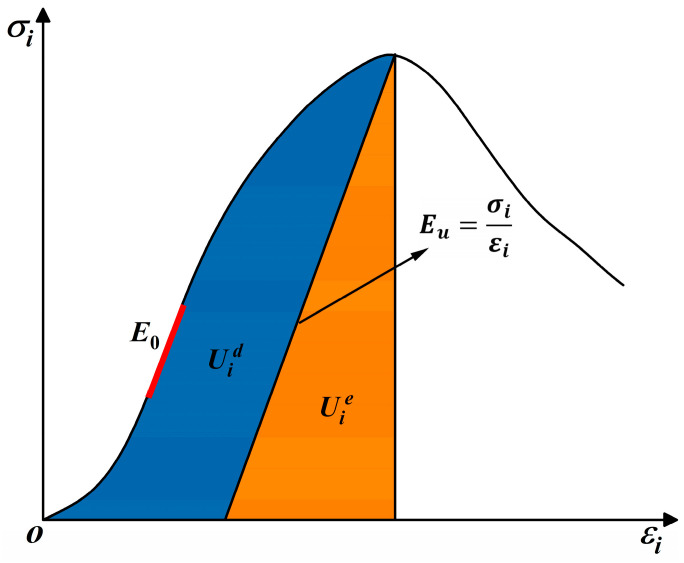
Energy calculation model.

Figure 1 shows the stress–strain curve of the rock mass unit. The blue area $U_{i}^{d}$ represents the energy dissipated by rock units due to internal damage and plastic deformation, and the orange area $U_{i}^{e}$ represents the releasable strain energy stored in the rock element. Under uniaxial compression conditions, only the maximum principal stress $\sigma_{1}$ is not zero; thus, we can obtain the calculation equation of $U_{e}\;$ and $U_{d}$.

(2) $$U=\int_{0}^{\varepsilon_{1}}\sigma_{1}d\varepsilon_{1}=\sum_{i=0}^{n}\frac{1}{2}\left(\sigma_{1i}+\sigma_{1i+1}\right)\left(\varepsilon_{1i+1}-\varepsilon_{1i}\right)$$

(3) $$U_{e}=\frac{1}{2}\sigma_{1}\varepsilon_{1}=\frac{\sigma_{1}^{2}}{2E_{u}}\;\approx \;\frac{\sigma_{1}^{2}}{2E_{0}}$$

(4) $$U_{d}=U-U_{e}$$

where $\sigma_{1}$ and ${\;\varepsilon }_{1}\;$ represent the maximum principal stress and the principal strain along the direction of the principal stress, respectively. $\sigma_{i}$ and ${\;\varepsilon }_{i}\;$represent the stress and strain values, respectively, at any position on the stress–strain curve. $E_{u}\;$ is the unloading elastic modulus, and in this study, we used the initial elastic modulus ${\;E}_{0}\;$ to replace it for calculation [36].

**Figure 2:**
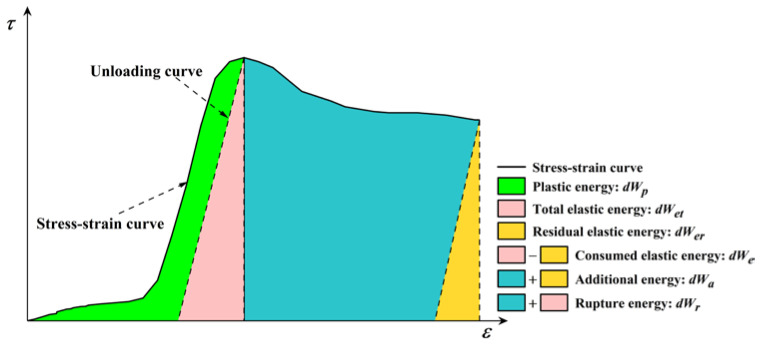
Typical shear stress–strain curve and associated energy characteristics for sample interfaces.

Nevertheless, this paper mainly focuses on the energy evolution characteristics of sandstone–concrete interface shear tests under different normal stress conditions. During the shear failure process of the sample, the sample accumulates energy in the form of elastic energy, on the one hand, and dissipates the energy to cause the formation of shear plane cracks, on the other hand, leading to shear failure and the formation of irrecoverable plastic deformation [37]. Therefore, based on the energy analysis method described earlier, the complete shear stress–strain curve of the sample under normal stress in a direct shear test is obtained, as shown in Figure 2. This curve provides a detailed understanding of the deformation and energy evolution behavior of the sample, in which the deformation, failure, and energy characteristics of the interface can be observed. According to the characteristics of energy accumulation and release, the shear stress–strain curve can be divided into four regions, as seen in Figure 2.

As illustrated in the figure, ${\;dW}_{et}\;$ is the total elastic energy, which is the elastic recovery zone under the unloading curve, calculated by Equation (5); during the calculation, the slope of the line connecting the starting point and the ending point of the unloading curve is used to represent the shear modulus of the sample. ${dW}_{p}\;$ is the plastic strain energy in the pre-peak stage, which is the plastic energy consumed to produce new cracks. ${\;dW}_{e}\;$ is the consumed elastic energy and ${dW}_{er}\;$ is the residual elastic energy. $dW_{a}$ is the additional energy provided by the further rupture of the sample in the post-peak region. ${dW}_{r}\;$is the rupture energy and ${\;dW}_{t}\;$ is the total energy dissipated or consumed during the entire process of the sample shear test. The formulae for each parameter are shown in Equations (5)–(10).

(5) $$dW_{\mathrm{et}}=\frac{{\tau_{p}}^{2}}{2E}$$

(6) $$dW_{p}=\int_{0}^{\varepsilon_{p}}\tau d\varepsilon -dW_{\mathrm{et}}=\int_{0}^{\varepsilon_{p}}\tau d\varepsilon -\frac{1}{2E}\tau_{p}^{2}$$

(7) $$dW_{\mathrm{er}}=\frac{{\tau_{r}}^{2}}{2E}$$

(8) $$dW_{e}=dW_{et}-dW_{er}=\frac{1}{2E}\left(\tau_{p}^{2}-\tau_{r}^{2}\right)$$

(9) $$dW_{r}=dW_{et}+dW_{a}-dW_{er}=\frac{1}{2E}\left(\tau_{p}^{2}-\tau_{r}^{2}\right)+\int_{\varepsilon_{p}}^{\varepsilon_{r}}\tau d\varepsilon$$

(10) $$dW_{t}=dW_{p}+dW_{r}$$

where $\varepsilon_{p}\;$ is the shear strain at the peak point, $\varepsilon_{r}\;$ is the shear strain of the residual shear stress, ${\;\tau }_{p}\;$ is the peak shear stress, ${\;\tau }_{r}\;$ is the residual shear stress, and *E* is the shear modulus.

### 2.2. Test Materials and Sample Preparation

To investigate the influence of normal stress on the shear mechanical and fracture properties of the rock–concrete serrated interface, mined yellow sandstone was selected as the test material, with a uniaxial compressive strength of 60 MPa. The concrete material in this study had a standard strength of C30, consisting of cement, water, sand, and coarse aggregate. The proportions of each component are illustrated in Table 1. The cement used in the concrete was P·O 42.5 Portland cement and the particle size of the coarse aggregate was 3.5–14 mm.

**Figure 3:**
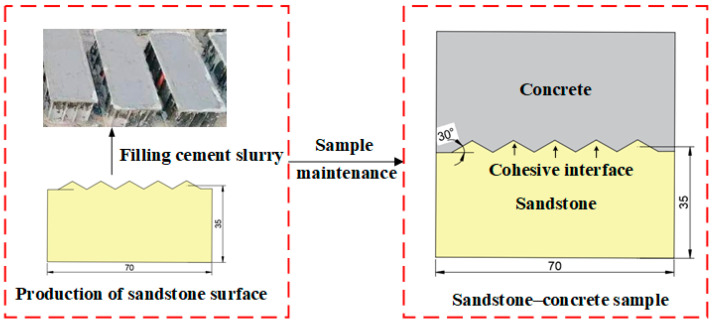
Sandstone–concrete sample preparation process.

After selecting the rock materials at the construction site, CNC water jet cutting was first used to prepare the required sandstone specimens with a size of 70 mm × 70 mm × 35 mm, as depicted in Figure 3. This high-accuracy processing method ensured that all specimens had consistent interfacial roughness, thus reducing the effect of roughness on their shear performance. Then, standard-sized molds were used to prepare sandstone–concrete specimens with dimensions of 70 mm × 70 mm × 70 mm. Before casting, molds were cleaned and oiled for better casting quality and more convenient demolding. The sandstone was first placed in one side of the molds, and then the concrete mixture was cast in the remaining space. The entire preparation process is presented in Figure 3. The specimens were demolded 24 h after being placed in a laboratory environment and were cured in a standard curing chamber for 28 days.

### 2.3. Test Systetm and Test Procedure

**Figure 4:**
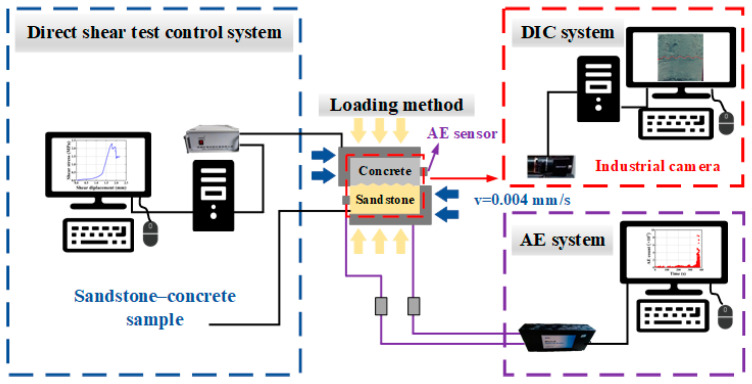
Schematic layout of the test system.

**Figure 5:**
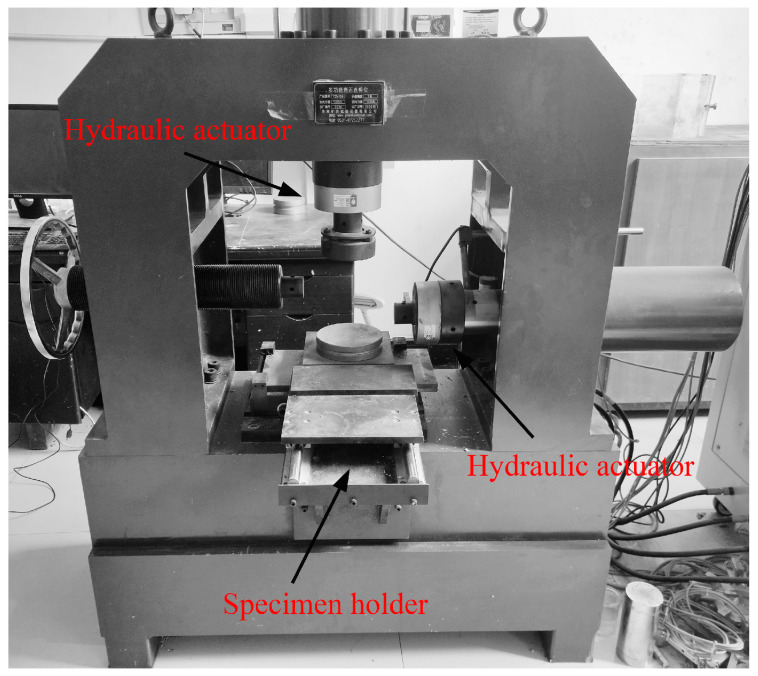
YZW100 multifunctional rock direct shear tester.

The test system in this paper was composed of a direct shear test control system, AE system, and DIC system, as shown in Figure 4. The shear apparatus in the control system was the YZW100 multifunctional rock direct shear tester (Jinan Ore–Rock Testing Instrument Co., Ltd., Jinan, China), as seen in Figure 5. The maximum normal and shear loads that the device could provide were both 500 kN. The machine operated through electro-hydraulic drive and could achieve constant stress and displacement control, which met the experimental conditions in this study.

The AE system involved the Micro-Ⅱ chassis (Physical Acoustics Corporation, America), two signal cables, two AE sensors, two preamplifiers, and the AE-win (AEwin32^TM^) software for capturing and processing the AE signals. For better signal acquisition, the threshold of the system and the amplification of the two preamplifiers were both 40 dB, and the sampling frequency was 10 MSPS (million samples per second). The charge-coupled device (CCD) camera (Basler/piA2400-17 gm) and the GOM Correlate software (GOM Correlate v8 SR1) were used in the DIC system. The former was used for image acquisition and storage and the latter was used for image processing and analysis. During the loading process, the frequency was 20 FPS (frames per second). To ensure the consistency of test data, all systems had to be simultaneously started in the test.

Before the shear test, all specimens were sprayed with paint to prepare the speckle pattern and two AE sensors were arranged diagonally behind the speckle surface of the specimen to acquire more data. The camera was adjusted to the optimal angle and a spotlight was also used for clearer and more detailed images.

In this paper, four groups of direct shear tests were conducted under various normal stress constraints. The normal stress σ was set at 0.2 MPa, 0.4 MPa, 0.6 MPa, and 0.8 MPa, respectively, and the loading rate was 0.004 mm/s under displacement control. To minimize potential errors and enhance the reliability of experimental results, three sandstone–concrete specimens were prepared for each group. 

## 3. Analysis of Test Results

### 3.1. Characteristics of Shear Displacement–Shear Stress Variation

**Figure 6:**
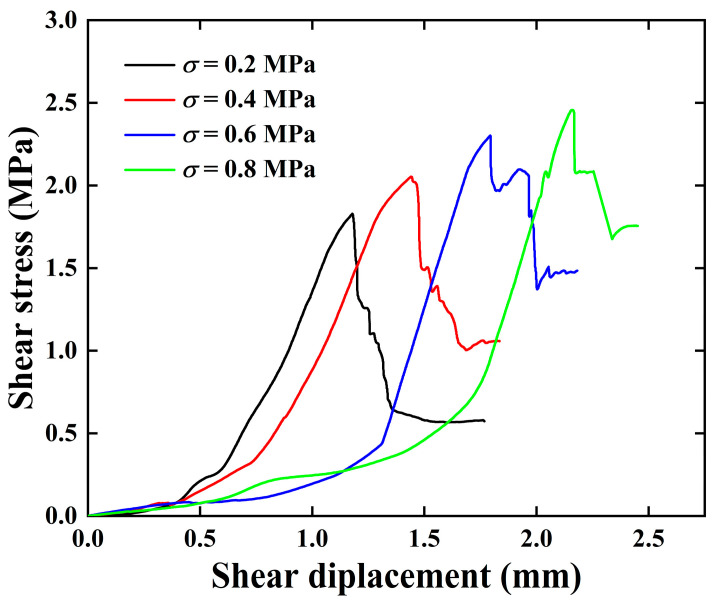
Shear displacement–shear stress curves under different stress conditions.

Given that the sandstone of uniform lithology used in this study underwent precise machining and was cast in standard molds alongside the same batch of concrete, the specimens exhibited identical surface characteristics. Consequently, the shear stress curves obtained from repeated tests under various stress levels demonstrated minimal discrepancies, with the evolution trends of these curves during the shearing process being largely consistent. Therefore, typical shear displacement–shear stress curves of the specimens from each case were selected for analysis in this section, as illustrated in Figure 6.

It can be seen from the curves that the deformation and failure process of the samples under different normal stresses is consistent, including the compaction stage, elastic stage, plastic stage, and failure stage. In the compaction stage, due to the existence of micro-cracks or pores between the interfaces, slow deformation of the sandstone–concrete interface occurs after the shear load is applied; the shear stress grows slowly with the increase in the shear displacement, and the interface’s shear stiffness is small. After a certain amount of coordination of deformation on the shear plane, the micro-cracks are sufficiently compacted, the sample enters the elastic stage, and the corresponding amount of shear displacement ranges from 0.5 to 1.5 mm. In the elastic stage, due to the micro-cracks or pores between interfaces and within the sample having been compacted, the sample is similar to the elastomer with relatively stable mechanical behavior, so the increase in shear stress with the increase in the shear displacement is approximately linear. When the shear stress is close to the peak, the curve presents nonlinear characteristics, meaning that the sample enters the plastic stage and micro-cracks within the sample are generated and propagate through the sample under the action of shear stress; when the shear stress reaches the interface’s shearing strength, that is, the peak of the curve, failure of the sample occurs, and the shear stress rapidly decreases. Under different normal stress levels, the rate of shear stress decay varies, with the sample having a higher shear modulus exhibiting a more rapid attenuation of shear stress. It can be observed that as the normal stress increases, the shear strength of the specimens continues to rise, and the shear displacement at the peak point progressively augments. When σ = 0.6 and 0.8 MPa, the curves show multiple stress drops in the vicinity of the peak and in the post-peak region, and the failure mode of the sample changes to progressive failure.

**Figure 7:**
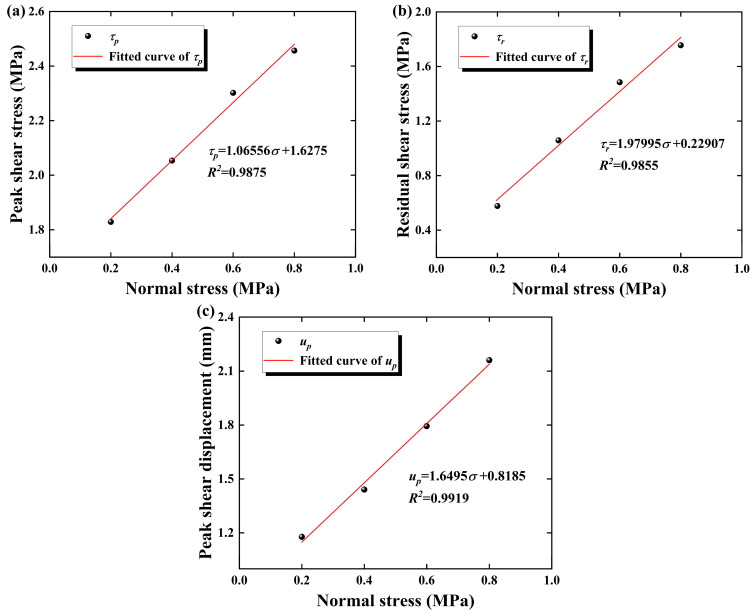
Variations in stress and strain characteristic values: (**a**–**c**) represent peak shear stress, residual shear stress, and peak shear displacement, respectively.

Table 2 presents the results from the direct shear tests conducted on sandstone–concrete specimens under different normal stress levels. Based on the data in Table 2, the variation trends of the characteristic values of stress and strain for the specimens are plotted in Figure 7. As shown in Figure 7a,b, the peak shear stress $\tau_{p}\;$ and residual shear stress $\tau_{r}\;$ of the specimens increase linearly with the increase in σ, which is consistent with the Mohr–Coulomb criterion [38]. Compared to the conditions at σ= 0.2 MPa, when σ is increased to the range of 0.4~0.8 MPa, the $\tau_{p}\;$ and $\tau_{r}\;$ of the specimens increases by 12.3~34.34% and 83.22~203.81%, respectively. This indicates that an increase in normal stress not only enhances the shear strength of the sandstone–concrete interface but also augments the sliding friction effect between the sandstone–concrete interfaces after shear failure. Consequently, the residual shear strength of the specimens also exhibits a trend of increasing with the normal stress. Notably, the impact of normal stress on the residual shear strength of the specimens is more pronounced, which is consistent with the experiment phenomenon of Liu et al. [39].

As can be seen from Figure 7c, the peak shear displacement $u_{p}$ of the specimen increases with increasing normal stress, σ. This is because the increase in σ inhibits the coalescence and interconnection of micro-cracks within the sandstone–concrete sample, resulting in an increase in the number of micro-fractures required to form macroscopic failure in the specimen. Consequently, the deformation of the specimen before failure is augmented.

**Figure 8:**
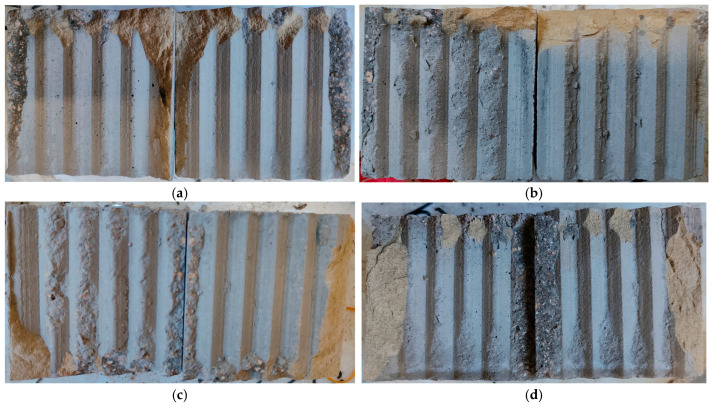
Typical shear failure surfaces of sandstone–concrete samples: (**a**–**d**) represent 0.2, 0.4, 0.6, and 0.8 MPa, respectively.

Figure 8 presents the typical shear failure surfaces of the sandstone–concrete samples under different normal stresses. Observations from the figure reveal that the serrations on the sandstone–concrete interface surface exhibit varying degrees of damage after shearing, with normal stress having a significant impact on interface deterioration. When the normal stress is relatively low, some serrations remain relatively intact, with shear damage predominantly occurring on the upper side. Conversely, under higher normal stresses, tooth tip abrasion occurs at the interface, and as the stress increases, the degree of serration damage intensifies, transitioning from the tooth tip to the tooth root. Furthermore, high stresses stabilize the debris filling between serrations, enhancing the shear resistance of the interface. In other words, as the normal stress increases, the peak failure strength also rises, which is consistent with the previous analysis.

### 3.2. Energy Evolution Characteristics

#### 3.2.1. Complete Energy Evolution Process Under Different Normal Stresses

**Figure 9:**
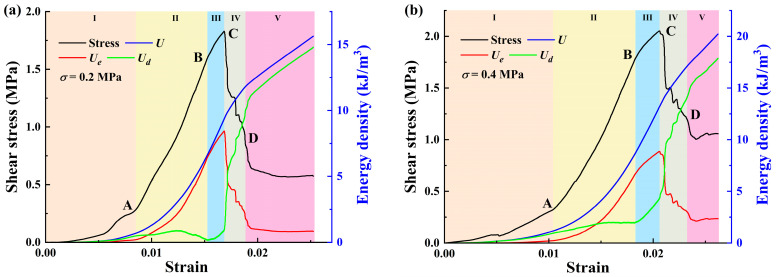

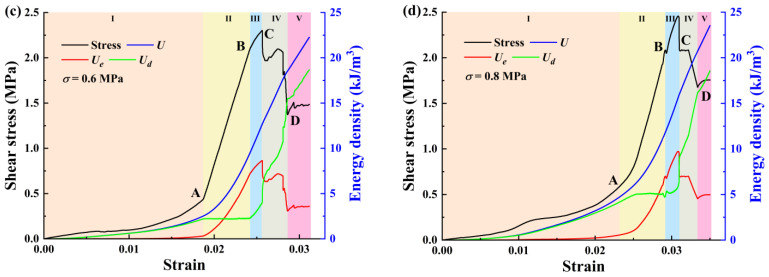
Complete energy evolution curves of samples under different normal stress levels: (**a**–**d**) represent 0.2, 0.4, 0.6, and 0.8 MPa, respectively.

During the sandstone–concrete interface shear tests, crack formation was accompanied by energy dissipation, which is an evolution process of micro-crack nucleation, propagation, coalescence, and failure [40]. From Figure 6, we obtained the stress–strain data during the shear process of the samples and then substituted them into Equations (2)–(4) to acquire three kinds of energy density data under different normal stress levels. Subsequently, these data were used to draw the complete energy evolution curves of the samples during the shear deformation and failure process under different normal stresses, as depicted in Figure 9.

The overall comparison shows that the characteristics of the energy evolution process under different normal stresses exhibit certain similarities. In view of the research from Chen et al. [41] about structural plane energy evolution during the shear process and our experimental results, the energy evolution process of the samples can be separated into five stages: the (I) compaction stage; (II) elastic deformation stage; (III) plastic stage; (IV) failure stage; and (V) post-peak degradation stage. In this study, the variations in the slopes of the stress–strain curves, namely Young’s modulus, and different strain energies are used to determine the transition points between different stages.

(1) Compaction stage (I): In this stage, the shear stress level of the sample is low. The total energy input into the sample, the elastic energy stored in the sample, and the energy dissipated by the sample to overcome the work done by the external load are all at a low level. To be specific, the total energy U basically coincides with the dissipative energy $U_{d}$ and maintains a stable and slow increase, while the elastic energy $U_{e}\;$is basically zero. This is mainly due to the shear densification in this stage, in which the input energy is mainly converted into dissipated energy for the closure of primary pores inside the sample. Note that the U and $U_{d}$ curves begin to diverge at the end of this stage (point A) and the elastic energy $U_{e}\;$ slightly increases, which means the voids between interfaces are healed and a small amount of elastic energy is generated for storage.
(2) Elastic deformation stage (II): After being compacted, the sample approaches a continuous medium, and shows obvious linear elastic characteristics. The total input energy density and elastic energy density are rapidly increasing, and basically maintain a parallel trend, while the dissipated energy density increases relatively slowly. This indicates that the energy absorbed by the sandstone–concrete specimens from the external environment is mainly stored in the specimens in the form of elastic strain energy.
(3) Plastic stage (III): The shear stress–strain curve of the specimen shows nonlinear characteristics in this stage, and the specimen experiences irreversible plastic deformation. The energy absorbed by the sample is mainly converted into dissipated energy for the propagation of internal cracks and micro-fractures. The proportion of dissipated energy increases, while the proportion of elastic strain energy decreases. Moreover, the elastic strain energy reaches its maximum value at the stress peak (point C). And the value of $U_{e}$ at this point is considered as energy storage limit of the sample. It should be noted that the elastic strain energies at point C under different conditions are 8.4419 kJ/m^3^, 8.6534 kJ/m^3^, 8.8720 kJ/m^3^, and 9.7226 kJ/m^3^, respectively, showing an increasing trend with the normal stress. This indicates that the growth of normal stress improves the capacity of specimens for energy storage and increases the energy required for crack development and propagation, thus enhancing the specimen’s bearing capacity.
(4) Failure stage (IV): The shear stress experiences a sudden drop and the micro-cracks inside the sample extend and coalesce into macroscopic cracks at point C, signifying that the sample has undergone structural instability and failure. The macroscopic cracks slide along the fracture surface, resulting in shear deformation. As the deformation increases, the total input energy continues to increase, while the stored elastic energy rapidly releases, and the dissipated energy density suddenly rises and then increases rapidly for crack aggregation, development, and coalescence. From the figures, the total input energy U of specimens before failure for each case are 9.3122 kJ/m^3^, 12.6147 kJ/m^3^, 13.1233 kJ/m^3^, and 15.6145 kJ/m^3^, respectively. Compared to the case when σ is 0.2 MPa, the total energy U has increased by 35.46%, 40.93%, and 67.68%, respectively. It shows that as the normal stress increases, the input energy required for specimen failure keeps increasing.
(5) Post-peak degradation stage (V): In this stage, as shear strain increases, shear stress begins to decrease, showing the characteristics of strain softening; the elastic energy density remains stable, while the dissipated energy and residual total energy show a parallel trend of steady growth. This is due to the slip friction between interfaces that causes the majority of the input energy to be converted into dissipated energy.

#### 3.2.2. Energy Evolution Analysis Based on Shear Strength

The shear energy of the structural plane evolves with shear deformation and micro-crack development. By analyzing the relationship between energy evolution and shear strength, we can deepen our understanding of energy dissipation processes, such as shear slip and shear rockburst, and specifically recognize the energy level of shear failure, facilitating the guidance of shear failure problems in engineering.

**Figure 10:**
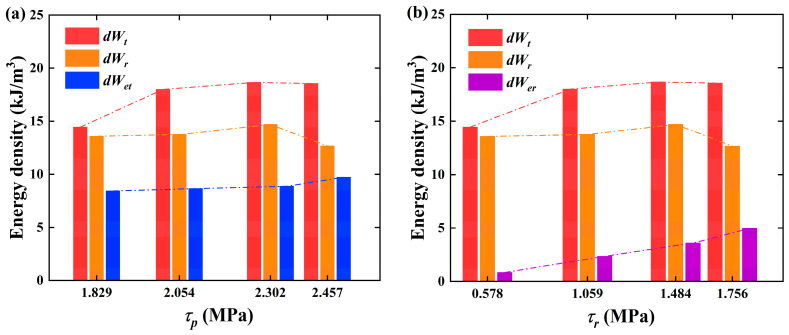
Relationship between shear energy and shear strength: (**a**,**b**) represent ${\;\tau }_{p}\;$ and $\tau_{r}$, respectively.

Previous studies have primarily focused on the relationship between strength and energy during the pre-peak stage [42], with relatively limited research on the relationship between strength and energy during the residual stage. Therefore, this section presents a statistical analysis of the total dissipated energy ${dW}_{t}$, total elastic energy ${\;dW}_{et}$, rupture energy ${\;dW}_{r}$, and residual elastic energy ${dW}_{er}\;$ for all the samples, as shown in Table 3. Combined with ${\;\tau }_{p}\;$ and $\tau_{r}$, provided in Table 2, a bar chart of energy density was plotted, as shown in Figure 10, illustrating the correlation between shear energy and strength characteristics. It can be observed that ${dW}_{t}$, ${dW}_{r}$ and ${dW}_{et}$ are closely related to the shear behaviors [43], and as evident from Equations (5)–(9), the shear failure energy is determined by the shear strength and the shear displacement. When the shear strength is relatively high, the structural plane exhibits greater resistance to shear stress, making it more difficult for surface asperities to undergo failure, which results in an increase in the shear failure energy. However, the rupture energy ${dW}_{r}$ exhibits a trend of initially increasing and then decreasing as the shear strength increases. This phenomenon may be attributed to the significant reduction in ductility during the post-peak failure stage of the specimens under the condition of 0.8 MPa compared to those under 0.6 MPa. This could be because the experiment was ended too early, so the ductility of the specimen was not well demonstrated. Consequently, the additional energy ${dW}_{a}$ required decreases correspondingly, leading to a reduction in the rupture energy.

### 3.3. Brittleness Evaluation Based on the Energy Evolution Mechanism

Brittleness is a significant material property for investigating rock failure and, due to its importance to the safety of engineering structures, there are many existing brittleness indices for rocks. From the research results of Chen et al. [20], the mechanism of energy evolution is closely related to the process of rock failure. Therefore, in this paper, we mainly focus on energy-based brittleness evaluation methods. In addition, in order to consider both the pre-peak and post-peak failure characteristics of the sandstone–concrete samples based on the complete stress–strain curves, we adopted the brittleness index ${BI}_{12}$ (Equation (11)) proposed by Kivi et al. [44] for the brittleness evaluation of the rock samples used in this test.

(11) $${BI}_{12}=\frac{1}{2}\left(BI_{1}+BI_{2}\right)=\frac{1}{2}\left(\frac{dW_{e}}{dW_{r}}+\frac{dW_{e}}{dW_{et}+dW_{p}}\right)$$

where $BI_{1}\;$determines the degree to which the fracturing process occurs in a self-sustaining manner and $BI_{2}\;$represents the proportion of the total input energy in the pre-peak region that is consumed during the post-peak failure process. Both of them vary in the range from 0 to 1, and for *B*$I_{1}$, a value of unity suggests a fully plastic state, while $BI_{2}\;$ indicates an increasing trend of brittleness from 0 to 1. Higher values of $BI_{1}$ and $BI_{2}\;$indicate higher ${BI}_{12}$, showing higher degrees of brittleness.

**Figure 11:**
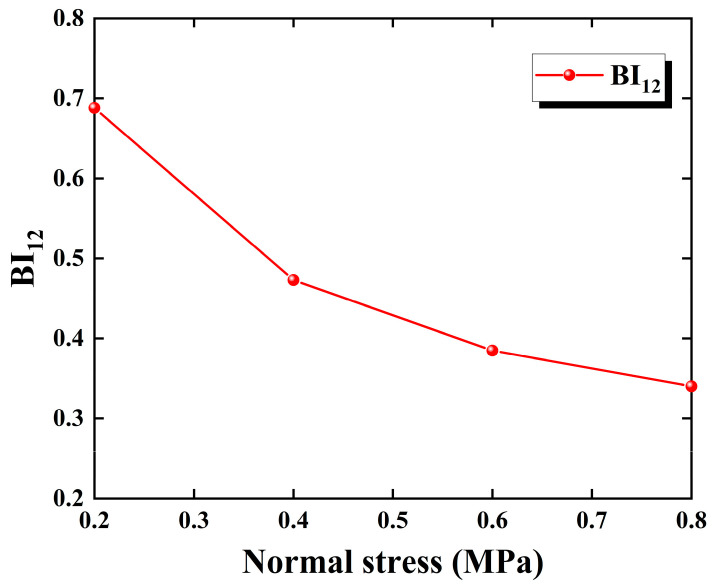
Variation in brittleness index ${\;BI}_{12}\;$ of samples under different normal stresses.

Based on the test data and Equation (11), the variation of ${BI}_{12}\;$with normal stress for the sandstone–concrete samples is obtained as shown in Figure 11. It can be seen from the figure that the brittleness of the samples under different conditions exhibits an overall decreasing trend with the increase in normal stress. And the results of the quantitative brittleness evaluation based on the index ${BI}_{12}\;$ effectively verifies the correctness of the qualitative analysis of the sandstone–concrete samples’ stress–strain characteristics and failure surfaces in the earlier part of the article.

## 4. Analysis of AE Characteristics and Fracture Behavior

### 4.1. Analysis of b Value

The prediction of rock failure characteristics using acoustic emission is currently a hot topic in the field of rock mechanics. Moreover, numerous scholars have conducted in-depth explorations of the laws of the acoustic emission *b* value. They consider that studying *b* values will enable us to gain insights into the rock failure mechanism, and it is of great practical significance for preventing the instability and failure of rock engineering projects [45,46]. In this study, the rock fracture behavior based on the *b* value theory is analyzed and discussed to further verify the accuracy of the analysis of the variation laws of energy storage and dissipation under different normal stress conditions.

The *b* value was initially mentioned in the Gutenberg and Richter rule proposed in 1936 [47], which presents the relationship between earthquake frequency and magnitude, and the formula is presented in Equation (12).

(12) lg⁡N =c – b×M

where M is the earthquake magnitude, N is the number of earthquakes in a slide time window, and c is an empirical constant.

Since then, some scholars have replaced magnitude with normalized AE amplitude to obtain the acoustic emission *b* value of rocks [48,49]. The above formula is thus modified as [49]

(13) $$\mathrm{lg}N(A_{0}/20)\;=c\;–\;b(A_{0}/20)$$

where $N(A_{0}/20)$ represents the cumulative AE frequency within the magnitude interval, $A_{0}$ is the AE amplitude, c is an empirical constant, and parameter b characterizes the level of AE activity.

Research has shown that as the *b* value increases, the AE activity increases slightly, and small-sized cracks occur inside the rock. Conversely, when the *b* value decreases, the AE activity becomes intense, and large-sized cracks and fractures appear inside the rock.

**Figure 12:**
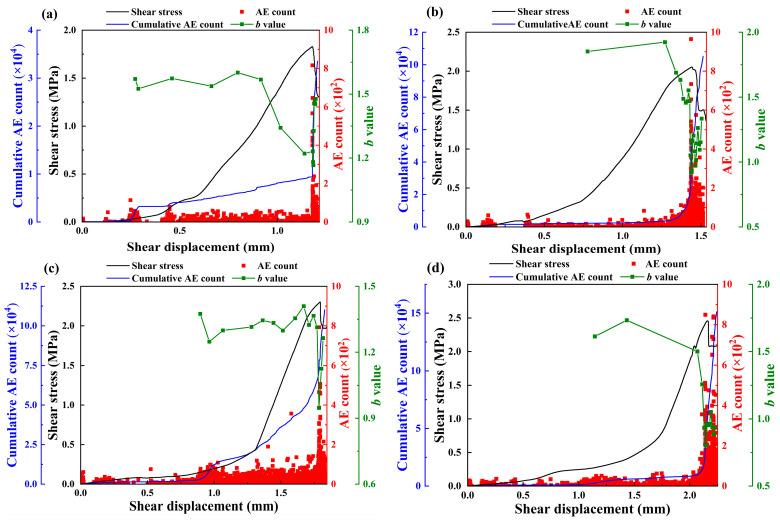
Characteristics of AE count and *b* value of samples under different normal stress levels: (**a**–**d**) represent 0.2, 0.4, 0.6, and 0.8 MPa, respectively.

The changing curves of the *b* value with the shear displacement of the samples under different normal stresses are depicted in Figure 12 (green). It can be seen from the figures that, during the entire loading process, the *b* value initially increases and then decreases in the stable crack propagation stage, and finally the *b* value fluctuates significantly at failure.

In the compaction stage, the primary pores and micro-cracks inside the sample begin to be compacted and closed, and the level of acoustic emission generated by the sample is relatively low. After the closure and compaction, the sample enters the elastic deformation stage, where there is little crack propagation activity observed, and the *b* value increases slightly. Then, the sample enters the stable crack propagation stage, where the micro-cracks begin to extend and coalesce, the large-sized cracks and fractures inside the sample also begin to increase, and the *b* value indicates a decreasing tendency. With the increase in shear displacement, the sample enters the non-steady crack propagation stage. The *b* value aggregates and fluctuates in this stage, and the large-sized cracks increase continuously. When the micro-cracks within the sample coalesce under shear loading to form a macroscopic crack, this signals a transition of the sample’s damage state from micro-plastic damage to macroscopic structural damage. At this point, the AE count increases sharply and the AE level during the subsequent loading process is notably elevated. Correspondingly, the *b* value plunges suddenly upon the initiation of the macroscopic crack, suggesting that the *b* value can reflect the internal structural damage state of the material. The above analysis on the *b* value characteristics is consistent with the results given by Shi et al. [50] and Dong et al. [51], who showed that the AE *b* value could measure the relative relationships in crack propagation and variation and manifest the development trends of cracks at different scales. Combined with the variation laws of stress–strain and energy evolution, the effectiveness of employing the changing trend of the *b* value to analyze the crack evolution process of the samples during shear tests has been verified.

It can also be observed that as the normal stress increases, the fluctuation range of the *b* value shows a downward trend as a whole when the sample fails. Specifically, for the sample at σ= 0.2 MPa, the *b* value fluctuates from 1.1 to 1.5, while for the sample at σ= 0.8 MPa, the *b* value fluctuates from 0.8 to 1.0. This phenomenon indicates a higher proportion of tensile cracks being converted into shear cracks [52], thereby requiring more energy to dissipate. Additionally, it can be seen that with the increase in normal stress, the scale of cumulative AE counts of the samples increases accordingly. This also implies that the increase in the stress level can strengthen the AE activities, generate more cracks inside the sample, and have more apparent energy dissipation during the fracture process.

### 4.2. Analysis of Crack Classification Characteristics

**Figure 13:**
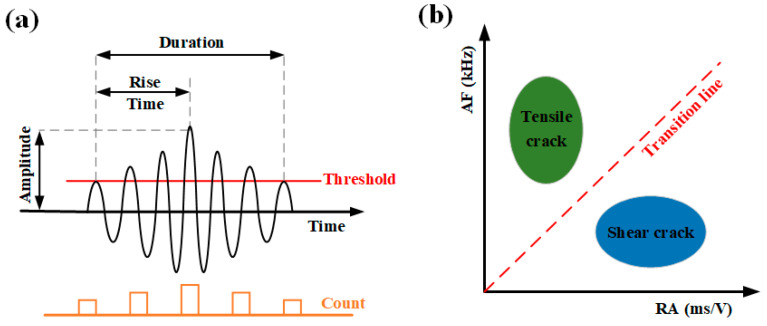
(**a**) Acoustic emission characteristic parameters and (**b**) crack classification method [54].

During the shearing process, stress concentration at the serrated interface leads to the initiation and subsequent propagation of cracks within the material. As important AE characteristic parameters, RA and AF are often used to investigate the fracture behaviors of rocks and classify the types of crack evolution in the fracture process [53]. Typically, during tensile failure, acoustic emission events show a smaller RA value and a larger AF value, whereas the opposite is true for shear failure [54,55]. The characteristic parameters and crack classification method are shown in Figure 13, and the calculation formula is shown in Equation (14).

(14) $$\left\{\begin{array}{c} RA=\frac{T_{RT}}{A_{0}}\\ AF=\frac{C_{AE}}{T_{D}} \end{array}\right.$$

where $T_{RT}\;$ is the rise time, $A_{0}\;$ is the amplitude, ${\;C}_{AE}\;$ is the acoustic emission count, and $T_{D}\;$ is the duration.

**Figure 14:**
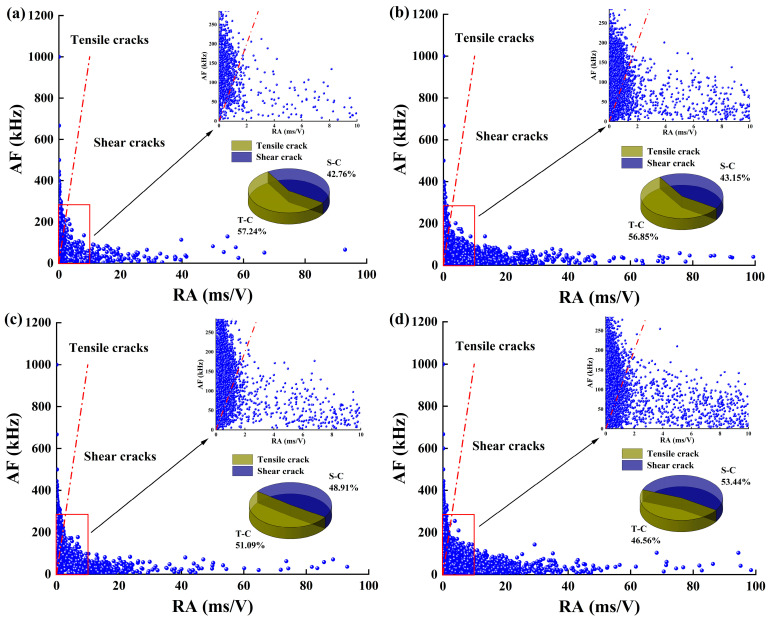
Crack classification and proportion: (**a**–**d**) represent 0.2, 0.4, 0.6, and 0.8 MPa, respectively.

Figure 14 shows the crack classification of the sandstone–concrete samples under different normal stresses. From the local magnification of Figure 14a–d, it can be seen that the point data on both sides of the RA and AF increase as the normal stress increases, and the number of points on the RA side increases more significantly. Upon integrating the analysis of the pie charts depicting the fractional distribution of RA and AF in each illustration, it is observed that as the normal stress increases, the fraction of shear cracks (S-C) within the sample’s crack evolution process progressively increases, while the fraction of tensile cracks (T-C) correspondingly decreases. This observation provides valuable insights into the impact of normal stress on the fracture mechanism within the rock–concrete materials. Among them, the percentage of shear cracks increases from 42.76% to 53.44%, and the percentage of tensile cracks decreases from 57.24% to 46.56%.

Under the conditions of 0.2–0.6 MPa normal stress, the fraction of tensile cracks is larger than for shear cracks, which indicates that tensile cracks dominate the failure process of the sample. When the normal stress is 0.8 MPa, the fraction of shear cracks is larger than tensile cracks, showing that the predominant mode of failure at this point is shear failure. The above analysis shows that in the direct shear tests of the rock–concrete interface, normal stress increases the number of cracks during the sample’s failure process. Additionally, there is a positive correlation between normal stress and the fraction of shear cracks, and a negative correlation with the fraction of tensile cracks. This phenomenon is in good consistency with the analysis of the *b* values.

### 4.3. Temporal Distribution Characteristics of Micro-Fracture Types in Samples

In this section, we correlate the acoustic emission waveform parameter values with the characteristics of the micro-fractures, and study the energy levels of shear fracture and tension fracture. The magnitude of the energy level can, to a certain extent, characterize the intensity of the rupture, and it is obtained by taking the decimal logarithm of the energy derived from acoustic emission techniques.

**Figure 15:**
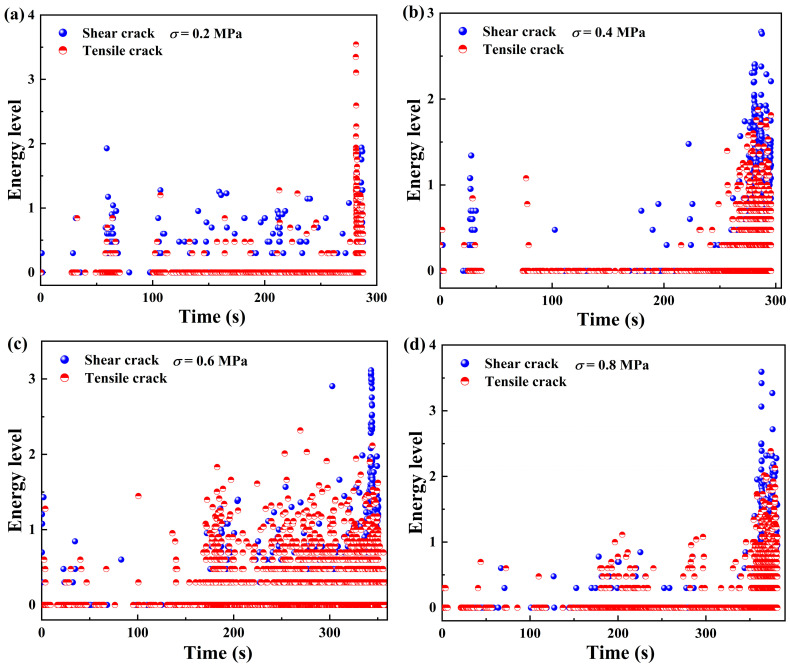
Temporal distribution characteristics of micro-fracture types in samples under different normal stress levels: (**a**–**d**) represent 0.2, 0.4, 0.6, and 0.8 MPa, respectively.

The temporal distribution characteristics of the micro-fracture types of the samples under different normal stresses are shown in Figure 15. It can be seen from the figure that the energy level of the micro-fractures is generally less than 1.5 energy levels in the early to mid stage of shear loading under the normal stress condition of 0.2 MPa, and as the sample approaches the failure stage, the number of micro-fractures continues to increase and the high-energy level fracture type is mainly tensile fracture. As shown in Figure 15a–d, with the increase in normal stress, the high-energy fracture type gradually changes from tensile fracture to shear fracture when approaching the failure stage. The energy level of both shear and tensile fracture increases, but the tensile fracture is mainly distributed below the second energy level. Similarly, the proportion of shear fracture increases with the increase in normal stress, which is consistent with the trend of shear and tensile cracks in Figure 14.

### 4.4. Analysis of Full-Field Strain

Through the digital image correlation algorithm, the DIC technique can obtain the global displacement field and strain field on the surface of the specimen during the loading process, and can thus reveal the strain localization and crack evolution process on the surface of the specimen. Therefore, in this section, we have acquired the full-field strain of all specimens under different normal stresses, as shown in Table 4, to investigate the failure modes of the sandstone–concrete specimens. Table 4 presents the strain evolution processes of all specimens under different conditions during the loading process that reflect the crack propagation patterns of all specimens.

In the shear-densification phase, varying degrees of strain appear at the tooth tips of the sawtooth interface under different normal stress conditions. Additionally, a more pronounced strain concentration phenomenon is observed on the right side of the sample. The first principal strain manifests as a tensile strain, varying from 0.05% to 0.9%, and it can be seen from the table that as the applied stress levels increase there is a notable increase in the number of wing-shaped cracks and a corresponding enlargement of the wing-shaped strain region. During the elastic phase, the strain region generated by the shear-densification process continues to grow, with a range of 0.95% to 2.35%. There is an observed enhancement in the wing-shaped strain concentration effect, which is primarily distributed at the intervals between the teeth of the serrated interface. The strain concentration at the right end is due to the concentration of shear stresses, and the increase in normal stress shows little influence on the strain concentration at the elastic stage of different groups of samples. After entering the plastic phase, the principal strain of the strain concentration at the right end continues to increase, resulting in the initial tensile wing-shaped cracks; different degrees of tensile wing-shaped cracks also appear at the inter-tooth areas of the sawtooth interface, and the strain range is 2.65–4.75%. The vacant areas of the figures in Table 4 are a result of significant macro-crack propagation, which has led to partial detachment of the samples’ surface. With the increasing shear displacement, the tensile wing-shaped cracks generated in the plastic phase are connected, finally leading to the failure of the sample and shear slip instability.

From the above analysis, it can be concluded that the propagation and penetration of tensile wing-shaped cracks are the leading causes of the sample failure. With the increase in normal stress, the tensile principal strain in the wing-shaped strain concentration zone increases, enhancing the samples’ resistance to shear deformation. In general, for the sandstone–concrete direct shear samples, instability occurs only on the direct shear plane, while the development of wing-shaped cracks is significantly affected by normal stress. As the normal stress increases, the development of wing-shaped cracks in the normal direction becomes constrained, and their propagation direction shifts towards the shear direction. The shear resistance provided by the interface serrations significantly impedes crack propagation until most of the serrations are sheared off, leading to the slip failure of the sample.

## 5. Conclusions

This paper first analyzes the characteristics of shear stress–displacement curves under different normal stress levels and discusses the failure surface morphologies of specimens. Then, based on energy calculation methods, the shear energy evolution mechanism is investigated, as well as its application in a brief brittleness evaluation. Finally, combined with AE and DIC techniques, the crack propagation and classification evolution process of the specimen are studied, and the fracture evolution behaviors of the sandstone–concrete interface are discussed. The main conclusions are as follows:

(1) The increase in normal stress inhibits the coalescence and penetration of micro-cracks within the sample, thereby enhancing the load-bearing capacity. The samples’ peak shear strength, residual strength, and shear displacement exhibit a linearly increasing relationship with normal stress.
(2) The energy evolution features during the failure process are discussed in detail and the impact of normal stress on energy evolution is also revealed. An increase in normal stress can increase the energy storage limit of the sample, as well as the energy required for crack extension and penetration. In addition, the brittleness evaluation model ${BI}_{12}$ indicates that the brittleness of the samples during direct shear tests shows a decreasing trend with the increase in the normal stress level.
(3) The analysis results of the *b* values show three-stage changing characteristics as the deformation increases in the shear process for specimens under different conditions. The *b* value initially increases, then decreases, and finally fluctuates significantly at failure. And it is closely consistent with the specimen’s crack propagation scale, from small to large.
(4) With an increase in normal stress, the failure type of the samples became predominantly shear failure, and the proportion of shear cracks increased from 42.76% to 53.44% while the proportion of tensile cracks decreased from 57.24% to 46.56%. On the eve of sample destabilization, the high-energy level fracture type gradually changes from tensile fracture to shear fracture with the increase in normal stress, and the proportion of shear fracture also increases.
(5) In terms of fracture evolution behaviors, the development of wing-shaped cracks is strongly influenced by normal stress. As the normal stress level increases, the interface failure becomes more severe, the tensile principal strain in the wing-shaped strain concentration zone increases, and the sample’s shear deformation resistance is enhanced.

This paper mainly focuses on the shear energy evolution analysis and fracture behaviors of sandstone–concrete samples under different normal stress conditions based on indoor tests with controlled temperature and humidity. However, it lacks consideration of more complex engineering conditions, such as cyclic loading, high temperature, freeze–thaw effects, dry–wet cycles, acid base environment, and the samples’ coupling conditions. Therefore, to investigate the damage and failure properties under complex conditions in the future research, it is advisable to choose more complicated experiments like cyclic shear tests, on the one hand, and present a comprehensive analysis by means of numerical research and theoretical derivation, on the other hand.

In addition, in this study, the impact of surface roughness and materials’ properties on the shear characteristics of rock–concrete interfaces are not yet investigated. It is thus proposed that further experiments are performed on different samples. Moreover, the research has limited methods of testing. It is also recommended that microscopic testing techniques like SEM and NMR are adopted to clarify in-depth the shear behavior of interfaces from microscopic perspectives.

## Figures and Tables

**Table 1:** Concrete mixture.

Ingredient	Ratio (kg/m^3^)
Cement	461
Water	175
Sand	512
Coarse aggregate	1252

**Table 2:** Direct shear test results of sandstone–concrete samples under different normal stresses.

Normal Stress σ (MPa)	Shear Modulus E (GPa)	$\mathrm{Peak}\;\mathrm{Shear}\;\mathrm{Stress}\;\tau_{p}$ (MPa)	$\mathrm{Peak}\;\mathrm{Shear}\;\mathrm{Displacement}\;u_{p}$ (mm)	$\mathrm{Residual}\;\mathrm{Shear}\;\mathrm{Stress}\;\tau_{r}$ (MPa)
0.2	0.198	1.829	1.178	0.578
0.4	0.238	2.054	1.441	1.059
0.6	0.306	2.302	1.794	1.484
0.8	0.310	2.457	2.160	1.756

**Table 3:** Energy density values of samples under different normal stress levels.

Normal Stress σ (MPa)	$\mathrm{Total}\;\mathrm{Dissipated}\;\mathrm{Energy}\;{dW}_{t}$ (kJ/m^3^)	$\mathrm{Rupture}\;\mathrm{Energy}\;{dW}_{r}$ (kJ/m^3^)	$\mathrm{Total}\;\mathrm{Elastic}\;\mathrm{Energy}\;{dW}_{et}$ (kJ/m^3^)	$\mathrm{Residual}\;\mathrm{Elastic}\;\mathrm{Energy}\;{dW}_{er}$ (kJ/m^3^)
0.2	14.447	13.576	8.442	0.842
0.4	18.004	13.752	8.653	2.357
0.6	18.661	14.701	8.872	3.599
0.8	18.560	12.668	9.723	4.965

**Table 4:** Full-field strain evolution of samples under different normal stress conditions: **I**–**IV** represent shear-densification phase, elastic phase, plastic phase, and failure phase, respectively.

Normal Stress	Different Phases of Strain Evolution				Legend
	I	II	III	IV	
0.2 MPa	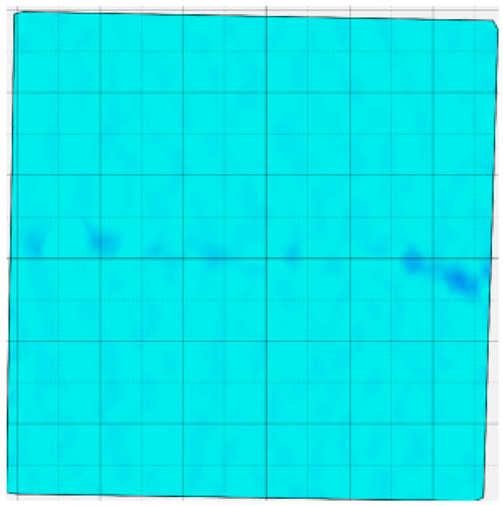	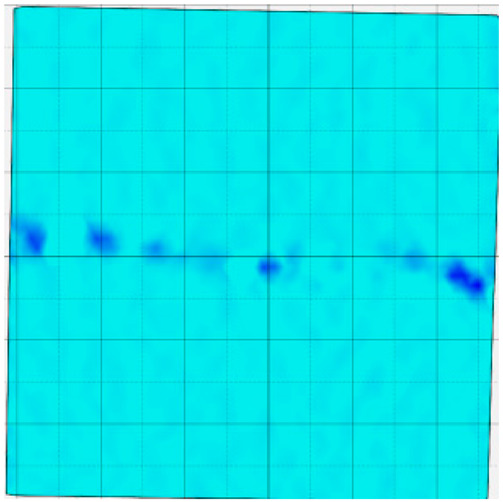	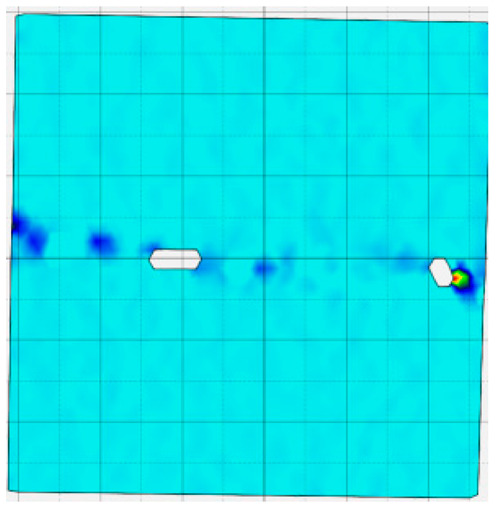	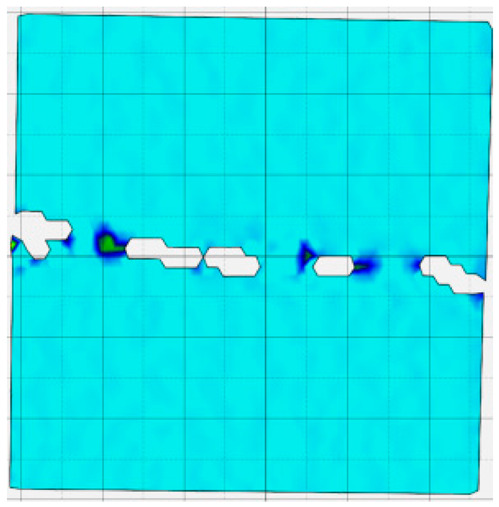	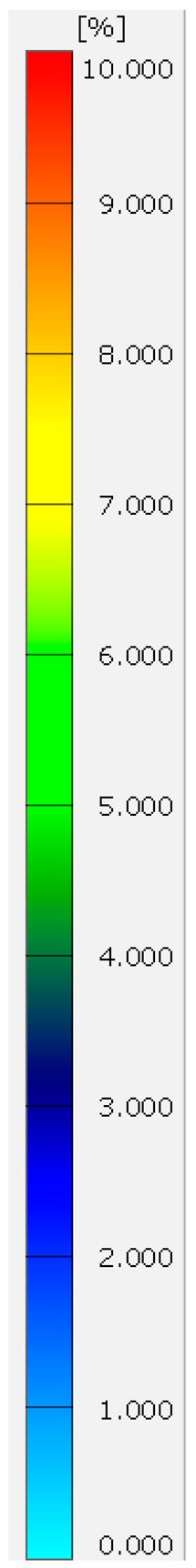
0.4 MPa	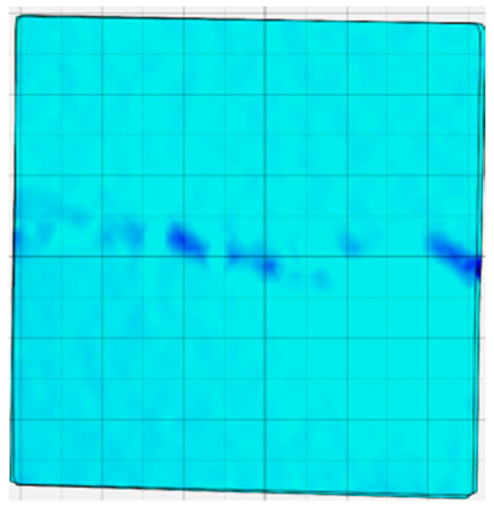	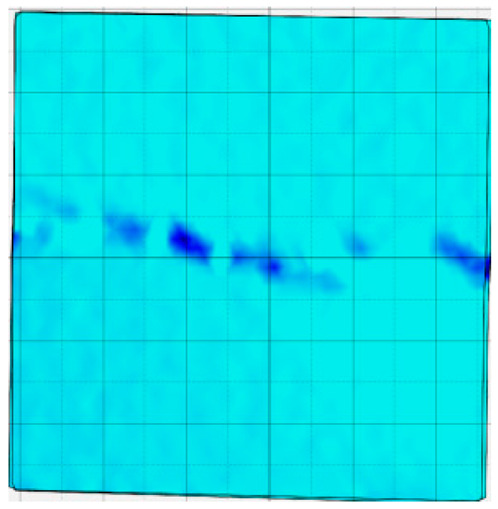	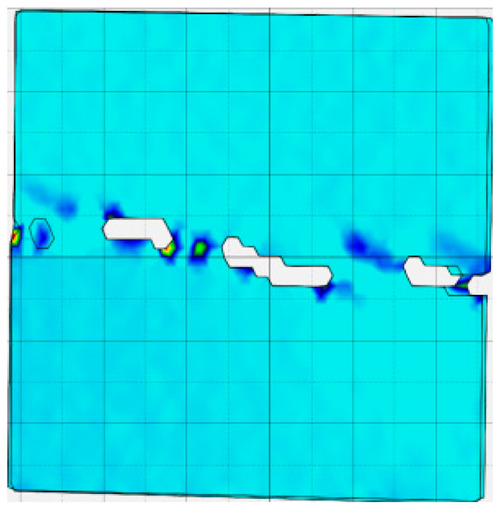	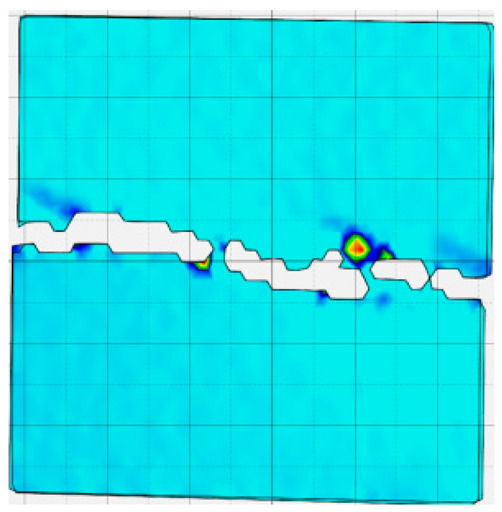	
0.6 MPa	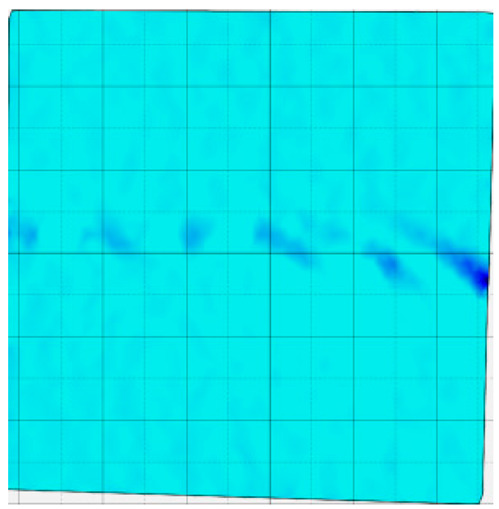	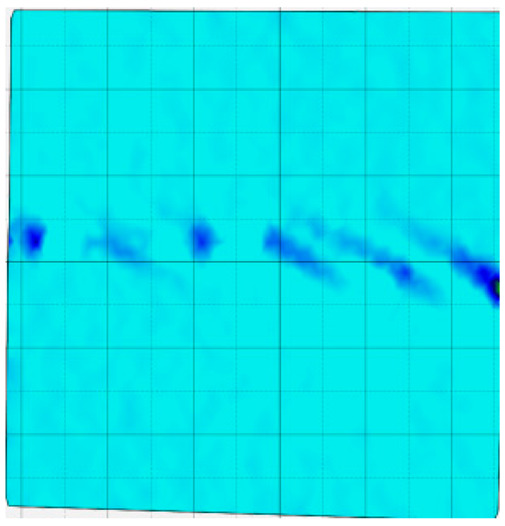	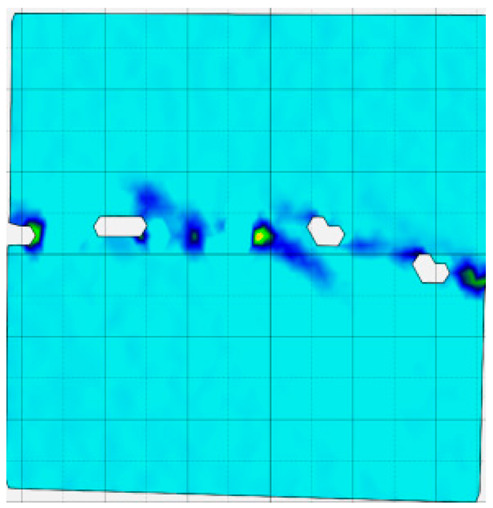	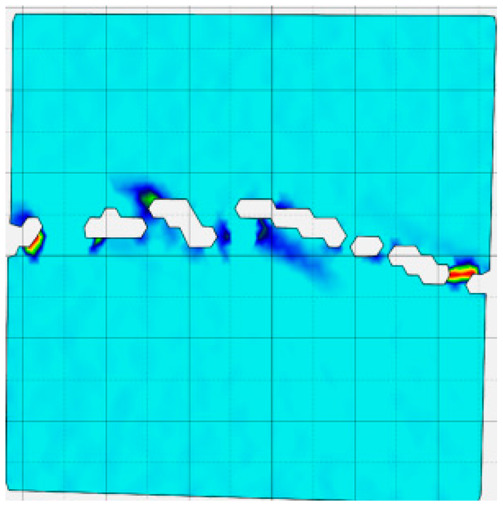	
0.8 MPa	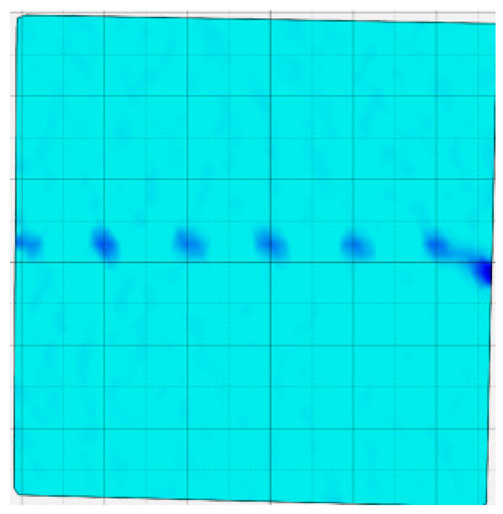	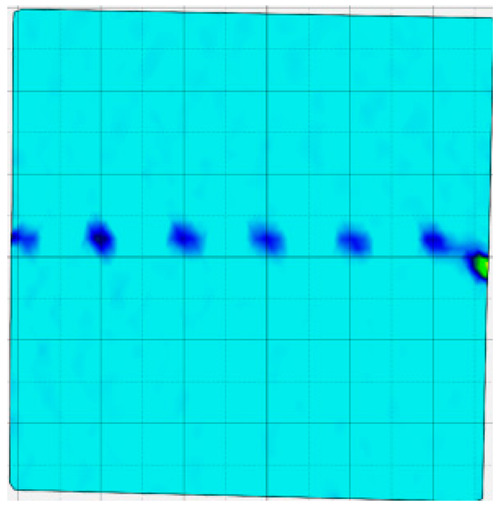	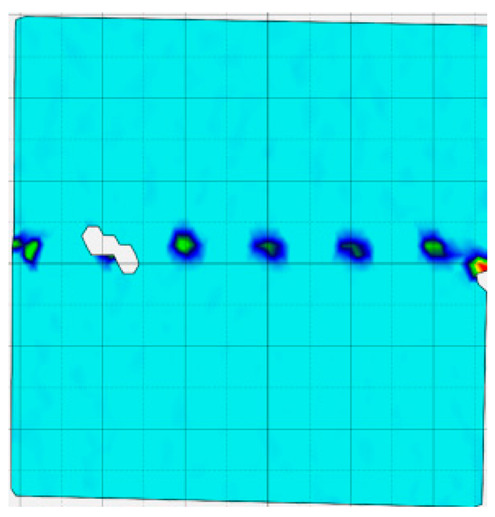	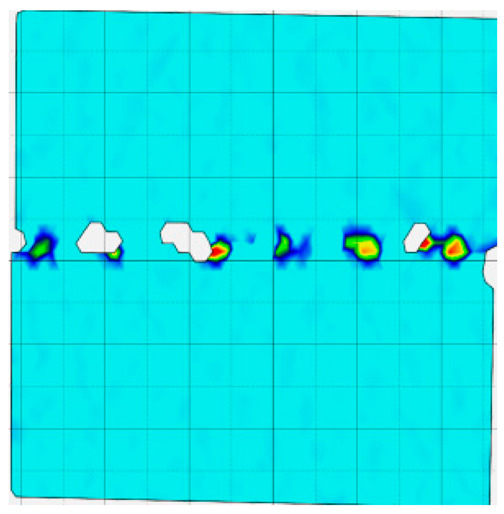	

## Data Availability

The original contributions presented in the study are included in the article; further inquiries can be directed to the corresponding author.

## Nomenclature

$A_{0}$	AE amplitude
AF	AE count/duration time
b	*b* value
${BI}_{12}$	brittleness index
$C_{AE}$	AE count
$dW_{a}$	additional energy
${dW}_{e}$	consumed elastic energy
${dW}_{er}$	residual elastic energy
${dW}_{et}$	total elastic energy
${dW}_{p}$	dissipated plastic energy in the pre-peak region
${dW}_{r}$	rupture energy
${dW}_{t}$	total dissipated energy
E	shear modulus
$E_{0}$	initial elastic modulus
$E_{u}$	unloading elastic modulus
M	AE threshold
RA	rise time/amplitude
$T_{D}$	AE duration time
$T_{RT}$	the rise time of AEs
U	total energy density
$U_{d}$	dissipated energy density
$U_{e}$	elastic energy density
$\varepsilon_{1}$	maximum principal strain
$\varepsilon_{i}$	strain value at any point
$\varepsilon_{p}$	shear strain at peak shear stress
$\varepsilon_{r}$	shear strain at residual shear stress
σ	normal stress
$\sigma_{1}$	maximum principal stress
$\sigma_{i}$	stress value at any point
$\tau_{p}$	peak shear stress
$\tau_{r}$	residual shear stress
$u_{p}$	peak shear displacement
